# Measuring Reliable Internet Connectivity Among Families with Children: Secondary Analysis of a US National Survey

**DOI:** 10.2196/69304

**Published:** 2025-08-08

**Authors:** Tran T Doan, Kelsey A Schweiberger, Samuel R Wittman, Tamar Krishnamurti, Sarah K Burns, Janel Hanmer, Kristin N Ray

**Affiliations:** 1 Department of Health Systems, Management, and Policy, Colorado School of Public Health, University of Colorado Anschutz Medical Campus, 13001 East 17th Place, Aurora, CO, 80045-2570, United States, 1 3037245050; 2 Department of Pediatrics, University of Pittsburgh School of Medicine, UPMC Children's Hospital of Pittsburgh, Pittsburgh, PA, United States; 3 Department of Medicine, University Pittsburgh School of Medicine, Pittsburgh, PA, United States

**Keywords:** child, parents, internet access, internet connectivity, broadband internet, telemedicine, telehealth, digital divide, health disparities

## Abstract

**Background:**

Reliable internet connectivity is crucial for family participation in pediatric digital health care, including telehealth. Lack of internet connectivity is a barrier to pediatric telehealth access. While surveys commonly inquire about metrics, such as internet plan or device ownership, fewer measures exist for the reliability of internet connectivity when needed. There is limited knowledge of the national prevalence of reliable internet connectivity among households with children and how reports of reliable internet connectivity are associated with use of internet plans and devices.

**Objective:**

We examined the prevalence of reliable internet connectivity among households with children and its association with digital technology access and sociodemographic factors.

**Methods:**

We performed a secondary data analysis of a US national cross-sectional survey examining parents’ health-seeking decisions for children younger than 18 years old. The respondent panel was hosted by the National Opinion Research Center (NORC) AmeriSpeak. This analysis focused on survey items on reliable internet connectivity, digital technology access (internet plan type and device ownership type), and sociodemographic characteristics (education, employment, geographic region, race and ethnicity, and disability) of parent respondents and their children. The dependent variable was a binary indicator of household reliable internet connectivity. Respondents were categorized as having unreliable internet connectivity if they self-reported internet worry or unreliable internet experience. Unadjusted Rao-Scott chi-square tests and adjusted multivariable logistic regressions with sampling weights were applied.

**Results:**

The final survey sample (N=1158) comprised 753 (55%) females, 614 (57%) non-Hispanic White, and 948 (81%) metropolitan respondents. There were 125 (12%) parents who reported internet worry, 152 (13%) parents who reported unreliable internet experience, and 76 (7%) parents who reported both. Combining these measures, we identified 201 (19%) parents with unreliable internet connectivity, defined as reporting either internet worry or unreliable internet experience. In contrast, 957 (81%) parents reported reliable internet connectivity in the household. In adjusted analysis, reliable internet connectivity was significantly associated with owning both nonmobile and mobile internet plans combined (86% reliable internet connectivity) versus nonmobile internet plan-only (67%; *P*=.001); postgraduate (94%) versus high school education (75%; *P*<.001); employment (84%) versus unemployment (76%; *P*=<.01); racial and ethnic marginalized status (77%) versus nonmarginalized (85%; *P*=<.01); and disability (70%) versus without disability (85%; *P*<.001), but not with device ownership, geographic region, race and ethnicity as separate groups, or parent sex.

**Conclusions:**

One-fifth of families with children experienced unreliable internet connectivity, highlighting an important dimension of the digital health divide that appears distinct from internet plan use or device ownership alone. Future research is needed to derive consensus on measuring reliable internet connectivity as a separate metric, including specifying the definition, survey questions, response options, and time frame of unreliability experience. Since reliable internet connectivity is needed for the growing field of digital health care, it is a critical issue for equitable pediatric health care access and delivery.

## Introduction

Pediatric telehealth use has proliferated since the COVID-19 pandemic but remains inequitable. About one-quarter of US parents report using telehealth for their child [1,2], but use is lower among households from low-income [3,4], rural [5], or marginalized racial and ethnic backgrounds [6,7]. Improving telehealth can help reduce disparities in access to child health services [8]. While internet connectivity is critical for family participation in pediatric digital health care, including telehealth [8,9], few estimates on reliable internet connectivity exist among households with children [7,10].

Most national surveys include metrics on internet plan or device ownership [2,11], overlooking questions on internet connectivity when needed. Since 2013, the US Census Bureau has asked whether individuals use the internet, how they access it, and about the smart devices they own [11]. Measuring only internet plan or device ownership is likely necessary but insufficient metrics to ensure that the internet is reliably available when people need it (eg, to keep telemedicine appointments).

Reliable internet connectivity might be an important dimension for improving family engagement in pediatric telehealth care. Accurately measuring reliable internet connectivity among families with children younger than 18 years is crucial, as parents play a key role in making medical decisions, directly impacting the accessibility of pediatric digital health care. In a US national sample of households with children from low-income only, parent respondents reported having some connectivity to the internet (90%), but half are under-connected without reliable high-speed internet [10]. This study did not survey households living above the federal poverty line. About 23% of families in a US Midwestern urban area reported having reliable high-speed internet [7]. Among these households, Black or Hispanic parents were less likely to report reliable high-speed internet than White parents [7]. There is limited knowledge of the national prevalence of reliable internet connectivity among sociodemographically diverse households, leading to a limited understanding of family under-connectedness and under-participation in pediatric telehealth.

This study examined the national prevalence of household reliable internet connectivity among US adults with children younger than 18 years and its associations of reliable internet connectivity with digital technology access and sociodemographic factors. We hypothesized that education and employment could have pathways to reliable internet connectivity through influence on family income and wealth as well as digital literacy and acceptability, which might impact the selection of an internet plan or frequency of updating devices, which could impact reliability beyond having a device or an internet plan. We hypothesized that race and ethnicity could influence reliable internet connectivity through structural racism occurring at the community or policy level. For example, digital redlining has left many neighborhoods with largely marginalized residents with less internet infrastructure [7,12-14]. We hypothesized that disability status could influence reliable internet connectivity, perhaps through the difficulty of navigating devices and applications that may not be designed with vision, hearing, or cognitive barriers in mind. Overall, measuring reliable internet connectivity could help identify worthwhile investments to reduce disparities in the use of pediatric telehealth and other digital health services for families through the maintenance of working devices or high-speed quality internet and alleviation of sociodemographic barriers to internet connectivity.

## Methods

### Study Design and Population

We performed a secondary analysis of cross-sectional survey data hosted by the National Opinion Research Center (NORC) AmeriSpeak, a probability-based panel of nationally representative US households [15]. This survey was developed to examine how parents decide to seek pediatric primary care services for their children sick with cold symptoms [1], but also inquired about telehealth use and internet connectivity. This survey was administered in February of 2022. Prior analyses of this survey examined the prevalence of primary care telemedicine use [1] and parent perspectives on telemedicine [16]. This analysis specifically focused on the prevalence of reliable internet connectivity among US households with children.

Respondents were eligible to participate if they reported being a parent or guardian of at least one child 0‐17 years old in the household and responsible for making medical decisions for their child. AmeriSpeak households were recruited into the panel by mail, telephone, and in-person field interviews. The survey was offered in English and Spanish and administered by phone and web modes. Respondents received a cash equivalent of US $5 for participation.

### Survey Instrument

The survey instrument included items about internet connectivity (self-reported internet worry and unreliable internet experience) and household digital technology access (internet plan type and device ownership type). Respondents completed sociodemographic survey items, including respondents' age, sex, race and ethnicity, disability, educational attainment, employment, metropolitan versus nonmetropolitan residence, US Census region, household income, and health literacy. They also reported children’s characteristics, including the child’s chronic health condition, the child’s insurance type, and the child’s primary care source. The survey language was also recorded.

Internet plan type and device ownership type were assessed through the American Community Survey items [17]. Respondents were asked, “Do you or any member of this household have access to the internet using a...?” and to select all that apply from a list of internet options: cellular data plan, broadband (high-speed) internet service, such as cable, fiber optic, or digital subscriber line service installed in this household, satellite internet service installed in this household, dial-up internet installed in this household, some other device, and none of these. Respondents also selected all that apply from a list of device options: desktop or laptop, smartphone, tablet or other portable computer, some other type of computer, and none of these.

Health literacy was evaluated through a single-item question, “How confident are you filling out medical forms by yourself?” and given options to choose from a 5-point Likert scale [18,19]. Disability status was evaluated through 3 standard questions adapted from the US Department of Health and Human Services [20]. Respondents indicated if any household adults responsible for children were blind or deaf or had a physical, mental, or emotional condition and were presented with Yes or No options.

Regarding child chronic health condition, respondents were asked, “Does your family have a child(ren) who has a chronic health condition that results in the use of or need for more services (eg, medical, mental health, and educational) than is usual for most children of the same age?” Regarding child primary care source, respondents answered, “Is there one particular place that you take your child(ren) for almost all their health care?” and “Is there one particular person that you think of as your child(ren)’s regular doctor or nurse?” Both survey items had options: Yes, No, or Unsure/I Don’t Know.

### Theoretical Framework

Our study was guided by Richardson et al [21] Framework for Digital Health Equity, which detailed the key multilevel digital determinants of health, including identifying opportunities to improve digital health equity at the individual, interpersonal, community, and societal levels. This framework expanded the National Institute on Minority Health and Health Disparities (NIMHD) Research Framework [22] by including a new domain of “digital environment.” Within the digital environment domain, this study was focused on digital determinants at the individual level through internet reliability and “digital technology access,” including individual internet plan type and device ownership type. We expected, in turn, that community level through determinants impacted individuals through “community infrastructure,” including community-level availability of cellular wireless and broadband access, quality, and affordability, which are differentially impacted by sociodemographic characteristics, such as parent education, employment, and geographic region; parent race and ethnicity; and parent disability. We proposed “reliable internet connectivity” as an additional component of individual-level technology access beyond internet plan and device ownership. Both technology access and community infrastructure were conceptualized as crucial for driving reliable internet connectivity.

### Covariates

This analysis focused on survey items related to internet connectivity, digital technology access, and sociodemographic characteristics of parent respondents and their children.

#### Dependent Variable

The dependent variable was a binary indicator of household reliable internet connectivity. Household respondents were categorized as having “unreliable internet connectivity” if they self-reported “always true” or “often true” to either survey items inquiring about “internet worry” or “unreliable internet experience.” All other respondents were categorized as having “reliable internet connectivity.”

Regarding internet worry, respondents answered, “In the last 12-months, I worried whether our internet access or data plan would run out or not be available when I really needed it.”

Regarding unreliable internet experience, respondents answered, “In the last 12-months, my internet connection was unreliable (such as too slow, poor sound, poor video quality, or video connection) when I really needed it.”

For both survey items, they chose from the options: always true, often true, sometimes true, or never true. These items were adapted from the validated 2-item food insecurity screening tool, asking if respondents had worried whether their food would run out or whether their food had run out within the past 12 months [23]. We adapted these items because they capture whether the respondent can count on a resource being available when they need it. While other survey items capture whether a respondent has a device or internet plan or whether they have digital literacy, we developed these 2 items to capture ideas of reliability versus unreliability of the resources, in this case, internet.

#### Independent Variables

Independent variables included household digital technology access, comprising internet plan type and device ownership type. We identified “nonmobile” internet plans as broadband, satellite, or dial-up. We then categorized households as having a nonmobile internet plan only, a mobile internet plan only (cellular data plan), both nonmobile and mobile plans combined, or neither. For device ownership, we identified households with a smartphone device only, a nonsmartphone device only (ie, desktop, laptop, tablet, portable computer, or some other type of computer), both smartphone and nonsmartphone devices combined, or neither.

For analysis, respondents’ employment was categorized as a paid employee or self-employed. Adequate health literacy was defined by respondents who reported “extremely” to the survey item on confidence in filling out medical forms [18,19]. Child’s insurance type through employers and direct purchases was grouped together, and federal plans (Medicaid, Medical Assistance, Tricare or other military health care, Indian Health Service, and Veterans Affairs) were grouped together. Based on responses to items about having a child’s primary provider and primary place of care, responses were categorized as having a regular place and provider, a regular place but not a regular provider, a regular provider but not a regular place, and no regular provider or place.

Respondents were categorized as belonging to a racial and ethnic marginalized group if they identified as Hispanic ethnicity or from Black, Asian, multiracial, or “other” racial backgrounds [24]. Non-Hispanic White individuals were considered to have racial and ethnic nonmarginalized status. Disability status was assigned if respondents reported a household adult being deaf, being blind, or experiencing difficulties related to a physical, mental, or emotional condition.

We examined missingness for key covariates within the data. Because we observed low levels of missingness (<4% of responses with any missingness), we did not impute data and instead conducted a complete case analysis for the 96% of survey responses with complete data for this analysis.

### Data Analysis

We applied descriptive statistics to describe household sociodemographic characteristics. Unadjusted bivariate analyses (Rao-Scott F chi-square tests) were conducted to calculate the prevalence of reliable internet connectivity in respondents overall and across household sociodemographic characteristics [1,12,25,26].

We then applied adjusted multivariable logistic regression to examine associations of reliable internet connectivity with specific clusters of variables chosen a priori to reflect digital technology access and sociodemographic characteristics. These characteristics were chosen for their potential association with reliable internet connectivity, including technological barriers (operationalized through internet plan type and device ownership type covariates) [2,27], financial (employment) [28], digital literacy (education) [28], geographic region (metropolitan vs non-metropolitan) [5,29,30], racial and ethnic marginalized status [7,31], and disability status [32,33].

Four regression models were applied to sequentially test these associations. The first model tested only the digital technology access variables (internet plan type and device ownership type), hypothesizing that duplicative internet plans and multiple devices would be associated with increased reliable internet connectivity. The second model tested the technology access as well as sociodemographic variables, with the hypothesis that higher educational attainment, employment, and metropolitan geographic regions would be associated with increased reliable internet connectivity. The third model tested technology access, sociodemographic variables, and racial or ethnic marginalized status, hypothesizing that marginalization would be associated with decreased reliable internet connectivity due to systemic disinvestment in marginalized communities due to structural racism. The final model tested technology access, sociodemographic variables, racial or ethnic marginalized status, and disability status, hypothesizing that living with a disability would be associated with decreased reliable internet connectivity due to a lack of accessibility of devices, applications, and internet service companies.

Our primary multivariate regression analyses used racial and ethnic marginalized status as a proxy for examining structural racism, but we also examined race and ethnicity as separate groups in sensitivity analysis. We tested alternative geographic variables in sensitivity analysis, replacing metropolitan status with four US Census regions (Northeast, Midwest, South, and West).

Predictive margins were then applied to generate adjusted proportions, representing the predicted prevalence of the outcome for a covariate level after adjusting for other covariates in the model. Model specifications were assessed using a link test and Hosmer-Lemeshow test. All data analyses applied sampling weights and dropped missing responses.

Final sampling weights were produced by computing NORC panel base sampling weights and study-specific base sampling weights raked to external US population totals [34]. Significance testing was performed using an α level of .05. Analyses were conducted using Stata version 17 (StataCorp, LLC).

### Ethical Considerations

Institutional Review Boards at the NORC at the University of Chicago and the University of Pittsburgh considered this study exempt from human participants review (number 23020197). The University of Pittsburgh research team received deidentified data only from NORC for survey analysis.

## Results

### Survey Sample Characteristics

Of 6015 panelists invited to participate in the survey, 1599 (27%) completed the eligibility screener. Of those who completed the screener, 1297 (81%) were considered eligible. Of those eligible, 1206 (93%) completed the survey. After dropping missing responses (n=48), the total sample size included in the analysis was 1158 parent respondents for a <4% missingness among those who completed the survey.

The survey sample comprised 753 (55%) females, 614 (57%) non-Hispanic Whites, and 948 (81%) metropolitan respondents (Table 1). There were 125 (12%) parents who reported internet worry, 152 (13%) who reported unreliable internet experience, and 76 (7%) who reported both. There were 201 (19%) parents who had unreliable internet connectivity, defined as reporting either internet worry or unreliable internet experience. In contrast, 957 (81%) parents had reliable internet connectivity. About 817 (70%) parents responded “never true” to either internet worry or unreliable internet experience.

**Table 1.** Respondent characteristics (AmeriSpeak Panel, parents of children aged 0‐17 years old (N=1158).

Characteristics	Sample size, n (weighted %)	SE
Respondent age (years)		
18‐29	151 (11.9)	1.3
30‐44	731 (59.2)	2.0
45‐59	248 (26.2)	1.9
≥60	28 (2.7)	0.8
Respondent sex		
Female	753 (55.2)	2.0
Male	405 (44.8)	2.0
Respondent race and ethnicity		
Hispanic	358 (22.2)	1.5
White, Non-Hispanic	614 (56.8)	2.0
Black, Non-Hispanic	103 (10.9)	1.3
Asian, Non-Hispanic	33 (6.6)	1.3
Multiracial or Other, Non-Hispanic	50 (3.6)	0.6
Respondent education		
Less than high school	66 (9.0)	1.3
High school graduate or equivalent	164 (24.0)	1.9
Some college or vocational school	427 (25.0)	1.5
Bachelor’s degree	307 (26.2)	1.7
Postgraduate study or professional degree	194 (15.7)	1.3
Respondent employment		
Yes	831 (70.9)	1.9
No	327 (29.1)	1.9
Household income, (US $)		
Less than 30,000	304 (27.2)	1.8
30,000 to less than 60,000	293 (22.9)	1.7
60,000 to less than 100,000	293 (24.3)	1.6
100,000 or more	268 (25.6)	1.7
Metropolitan status		
Metropolitan	948 (80.5)	1.6
Non-Metropolitan	210 (19.5)	1.6
Geographic region		
Northeast	156 (16.1)	1.7
Midwest	266 (20.9)	1.5
South	427 (38.7)	1.9
West	309 (24.3)	1.6
Respondent health literacy		
Yes	736 (60.8)	2.0
No	422 (39.2)	2.0
Survey language		
English	1092 (96.0)	0.7
Spanish	66 (4.0)	0.7
Respondent deaf or hard of hearing		
Yes	64 (6.3)	1.1
No	1094 (93.7)	1.1
Respondent blind or difficulty seeing		
Yes	107 (10.7)	1.4
No	1051 (89.3)	1.4
Respondent physical, mental, or emotional condition		
Yes	125 (11.7)	1.4
No	1033 (88.3)	1.4
Child with a chronic health condition		
Yes	207 (17.1)	1.5
No	900 (77.9)	1.6
Unsure	51 (5.0)	0.8
Child’s insurance type		
Through employer or purchased directlyy	622 (54.4)	2.0
Medicaid or other federal payer	505 (42.8)	2.0
Uninsured	31 (2.9)	0.7
Child’s primary care source		
Regular place and provider	846 (71.9)	1.7
Regular place but not provider	124 (11.3)	1.3
Regular provider but not place	48 (3.6)	0.7
No Regular place nor provider	140 (13.3)	1.8
Household internet plan type		
Mobile plan-only	172 (14.8)	1.4
Mobile and nonmobile plans	822 (70.0)	1.8
Nonmobile plan-only	147 (13.4)	1.4
None	17 (1.8)	0.6
Household device ownership type		
Smartphone device-only	170 (16.2)	1.5
Smartphone and nonsmartphone devices	875 (73.0)	1.8
Nonsmartphone device-only	94 (8.5)	1.1
None	19 (2.3)	0.7
Internet worry		
Yes	125 (12.0)	1.4
No	1033 (88.0)	1.4
Unreliable internet experience		
Yes	152 (13.2)	1.4
No	1006 (86.8)	1.4
Reliable internet connectivity		
Yes	957 (81.4)	1.7
No	201 (18.6)	1.7

### Unadjusted Bivariate Analyses

Reliable internet connectivity was the most prevalent among Asian (29/33, 91%) and White (537/614, 87%) respondents, and least prevalent among Black (80/103, 69%) and Hispanic (269/358, 71%) respondents (*P*<.001) (Table 2). Prevalence of reliable internet connectivity was highest among those with the highest educational attainment (182/194, 96%; *P*<.001) and households in the highest income bracket (251/268, 92%; *P*<.001). Reliable internet connectivity prevalence was higher among households with children insured by a private health plan (554/622, 89%) compared to a public health plan (379/505, 71%; *P*<.001).

**Table 2.** Unadjusted prevalence of reliable versus unreliable internet connectivity by respondent characteristics (N=1158).

Characteristics	Reliable internet connectivity, n (weighted %)	Unreliable internet connectivity, n (weighted %)	Design-based *P* value
Respondent age (years)			.36
18‐29	117 (74.9)	34 (25.2)	
30‐44	607 (82.2)	124 (17.8)	
45‐59	211 (83.8)	37 (16.3)	
≥60	22 (69.3)	6 (30.7)	
Respondent sex			<.01
Female	605 (76.8)	148 (23.2)	
Male	352 (87.0)	53 (13.0)	
Respondent race and ethnicity			<.001
Hispanic	269 (71.3)	89 (28.7)	
White, Non-Hispanic	537 (86.5)	77 (13.5)	
Black, Non-Hispanic	80 (69.4)	23 (30.7)	
Asian, Non-Hispanic	29 (90.9)	4 (9.2)	
Multiracial or Other, Non-Hispanic	42 (81.5)	8 (18.5)	
Respondent education			<.001
Less than high school	49 (73.4)	17 (26.7)	
High school graduate or equivalent	114 (69.0)	50 (31.0)	
Some college or vocational school	340 (79.2)	87 (20.8)	
Bachelor’s degree	272 (88.8)	35 (11.2)	
Postgraduate study or professional degree	182 (95.9)	12 (4.2)	
Respondent employment			<.001
Yes	711 (85.8)	120 (14.3)	
No	246 (70.7)	81 (29.3)	
Household income, US $			<.001
Less than 30,000	215 (67.6)	89 (32.4)	
30,000 to less than 60,000	236 (77.8)	57 (22.2)	
60,000 to less than 100,000	255 (88.6)	38 (11.5)	
100,000 or more	251 (92.3)	17 (7.7)	
Metropolitan status			.61
Metropolitan	788 (81.0)	160 (19.0)	
Nonmetropolitan	169 (82.9)	41 (17.1)	
Geographic region			.11
Northeast	130 (82.1)	26 (17.9)	
Midwest	232 (84.2)	34 (15.8)	
South	340 (76.3)	87 (23.7)	
West	255 (86.5)	54 (13.6)	
Adequate health literacy			.02
Yes	627 (84.5)	109 (15.5)	
No	330 (76.5)	92 (23.5)	
Respondent survey language			.01
English	912 (82.1)	180 (17.9)	
Spanish	45 (64.2)	21(35.8)	
Respondent deaf or hard of hearing			<.001
Yes	35 (51.6)	29 (48.4)	
No	922 (83.4)	172 (16.7)	
Respondent blind or difficulty seeing			<.001
Yes	65 (55.2)	42 (44.9)	
No	892 (84.5)	159 (15.5)	
Respondent physical, mental, or emotional condition			<.001
Yes	74 (53.1)	51 (47.0)	
No	883 (85.1)	150 (15.0)	
Child with a chronic health condition			<.01
Yes	154 (72.5)	53 (27.5)	
No	770 (84.2)	130 (15.8)	
Unsure	33 (67.3)	18 (32.7)	
Child’s insurance type			<.001
Through employer or purchased directly	554 (89.4)	68 (10.6)	
Medicaid or other federal payer	379 (70.8)	126 (29.2)	
Uninsured	24 (86.9)	7 (13.1)	
Child’s primary care source			.13
Regular place and provider	716 (83.6)	130 (16.4)	
Regular place but not provider	98 (73.3)	26 (26.7)	
Regular provider but not place	38 (74.2)	10 (25.8)	
No regular place nor provider	105 (78.0)	35 (22.0)	
Household internet plan type			<.001
Mobile and nonmobile plans	719 (87.0)	103 (13.0)	
Mobile plan-only	129 (71.0)	43 (29.0)	
Nonmobile plan-only	96 (63.6)	51 (36.4)	
None	13 (78.8)	45 (21.2)	
Household device ownership type			<.001
Smartphone and nonsmartphone devices	753 (85.3)	122 (14.7)	
Smartphone device-only	121 (66.7)	49 (33.3)	
Nonsmartphone device-only	71 (77.0)	23 (23.0)	
None	12 (74.9)	7 (25.1)	

Families who completed the survey in English (912/1092, 82%) were more likely to have reliable internet connectivity than those completing the survey in Spanish (45/66, 64%; *P*=.01). Parents with a disability (35/64, 52% to 65/107, 55%; *P*<.001) and children with a chronic health condition (154/207, 73%; *P*=<.01) were less likely to have reliable internet connectivity.

Regarding digital technology access, reliable internet connectivity was more prevalent among families with access to both a mobile internet plan and another type of internet plan combined (719/822, 87%), compared to mobile internet plan-only (129/172, 71%) or nonmobile internet plan-only (96/147, 64%; *P*<.001). It was also more prevalent among families with access to both a smartphone and another device type combined (753/875, 85%), compared to smartphone only (121/170, 67%) or another device type only (71/94, 77%; *P*<.001). While internet plan use and device ownership were associated with reliable internet connectivity in unadjusted analyses, we found that even among families with multiple internet plans or multiple devices, 103/822, 13% to 122/875, 15% still had unreliable internet connectivity. This result may be influenced by the relationship between unreliable internet connectivity and sociodemographic factors (education, employment, race or ethnicity, or disability).

### Adjusted Multivariable Logistic Regression Analyses

Through sequential adjusted regression models, we observed that internet plan use, education, employment, racial or ethnic marginalized status, and disability status were all significantly associated with reliable internet connectivity, while device ownership and metropolitan status were not significantly associated (Table 3). The link test and Hosmer-Lemeshow test showed no evidence of improper specification across all four models.

**Table 3.** Adjusted prevalence of respondents with reliable internet connectivity by digital technology access and sociodemographic variables.

	Model 1^a^		Model 2^b^		Model 3^c^		Model 4^d^	
Characteristics	Adjusted proportions (95% CI)	Design-based *P* value	Adjusted proportions (95% CI)	Design-based *P* value	Adjusted proportions (95% CI)	Design-based *P* value	Adjusted proportions (95% CI)	Design-based *P* value
Household internet plan type		<.001		<.001		<.001		.001
Mobile and Nonmobile plans	0.86 (0.83‐0.90)		0.86 (0.83‐0.90)		0.86 (0.82‐0.89)		0.86 (0.82‐0.89)	
Mobile plan-only	0.75 (0.67‐0.84)		0.77 (0.70‐0.85)		0.77 (0.70‐0.85)		0.76 (0.68‐0.83)	
Nonmobile plan-only	0.64 (0.51‐0.76)		0.64 (0.54‐0.74)		0.65 (0.54‐0.75)		0.67 (0.56‐0.77)	
None	0.82 (0.53‐1.00)		0.79 (0.50‐1.00)		0.79 (0.51‐1.00)		0.81 (0.57‐1.00)	
Household device ownership type		.08		.24		.19		.40
Smartphone and nonsmartphone devices	0.83 (0.79‐0.87)		0.82 (0.78‐0.86)		0.82 (0.78‐0.86)		0.81 (0.77‐0.86)	
Smartphone device-only	0.73 (0.65‐0.81)		0.76 (0.69‐0.83)		0.76 (0.69‐0.83)		0.78 (0.71‐0.85)	
Nonsmartphone device-only	0.85 (0.77‐0.94)		0.86 (0.79‐0.94)		0.87 (0.80‐0.94)		0.87 (0.80‐0.93)	
None	0.80 (0.52‐1.00)		0.86 (0.67‐1.00)		0.86 (0.67‐1.00)		0.82 (0.60‐1.00)	
Respondent education				<.001		<.001		<.001
Less than high school			0.79 (0.68‐0.91)		0.81 (0.70‐0.92)		0.84 (0.74‐0.93)	
High school graduate or equivalent			0.73 (0.65‐0.81)		0.74 (0.66‐0.81)		0.75 (0.69‐0.82)	
Some college or vocational school			0.79 (0.74‐0.83)		0.78 (0.73‐0.83)		0.78 (0.73‐0.83)	
Bachelor’s degree			0.87 (0.81‐0.93)		0.86 (0.81‐0.92)		0.84 (0.81‐0.91)	
Postgraduate Study/Professional Degree			0.95 (0.92‐0.98)		0.95 (0.91‐0.98)		0.94 (0.91‐0.98)	
Respondent employment				<.01		<.01		<.01
Yes			0.85 (0.81‐0.88)		0.85 (0.81‐0.88)		0.84 (0.81‐0.88)	
No			0.75 (0.69‐0.81)		0.75 (0.69‐0.81)		0.76 (0.70‐0.81)	
Metropolitan status				.25		.60		.60
Metropolitan			0.80 (0.77‐0.84)		0.81 (0.78‐0.84)		0.81 (0.78‐0.84)	
Nonmetropolitan			0.85 (0.79‐0.91)		0.83 (0.77‐0.89)		0.83 (0.76‐0.90)	
Respondent racial and ethnic marginalized status						.02		<.01
Yes					0.77 (0.73‐0.82)		0.77 (0.73‐0.81)	
No					0.85 (0.81‐0.89)		0.85 (0.81‐0.89)	
Respondent disability status								<.001
Yes							0.70 (0.62‐0.77)	
No							0.85 (0.82‐0.88)	

a Model 1 included technology access variables.

b Model 2 included technology access and sociodemographic variables.

c Model 3 included technology access, sociodemographic, and racial and ethnic marginalized status variables.

d Model 4 included technology access, sociodemographic, racial and ethnic marginalized status, and disability status variables.

Based on findings from the final regression model, the prevalence of reliable internet connectivity was higher among families with access to both nonmobile and mobile plans combined (86%), compared to a nonmobile plan only (67%; *P*=.001, Table 3). Prevalence was higher among respondents who attained postgraduate degrees (94%) compared to high school degrees (75%; *P*<.001). Prevalence was higher among those who were employed (84%) compared to those who were unemployed (76%; *P*=.007).

We found that families belonging to a racial and ethnic marginalized group (77%) were associated with decreased prevalence of reliable internet connectivity than those belonging to a nonmarginalized group (85%; *P*=.009), despite adjusting for digital technology access and other sociodemographic variables. We also found that parent respondents with a disability were less likely to have reliable internet connectivity (70%) compared to those without a disability (85%; *P*<.001).

In adjusted models, alternative tested variables showed that geographic region defined by the US Census region (instead of metropolitan status) was not significantly associated with reliable internet connectivity (Table S1 inMultimedia Appendix 1). Race or ethnicity as individual categories (instead of a grouped marginalized status) was also not significantly associated with reliable internet connectivity (Table S2 in Multimedia Appendix 1). Adding the sex variable produced poorly fitted models (not shown) [30].

## Discussion

### Principal Findings and Comparison With Previous Works

In a national survey, approximately 1 of 5 parent respondents with children <18 years old were under-connected as measured through unreliable internet connectivity experience in the past year. The national prevalence of unreliable internet connectivity is higher among households with nonmobile internet plans only, decreased educational attainment, unemployed status, racial and ethnic marginalized status, and disability status. In contrast, approximately 4 of 5 households with children experienced reliable internet connectivity. Reliable internet connectivity was significantly associated with internet plan type and sociodemographic factors, including education, employment, racial and ethnic marginalized status, and disability status. These associations with both the internet plan type and sociodemographic factors further illustrate the complexity of measuring and enabling reliable internet connectivity, which could be leveraged to enhance pediatric digital health care access.

While internet plan type and device ownership type metrics are often studied, they are imperfect proxies of reliable internet connectivity. In bivariate unadjusted analyses, we found that even among families with multiple internet plans or devices, 13%‐15% of parent respondents still experienced internet unreliability. Our findings showed that internet plan type and device ownership do not perfectly predict the presence or absence of reliable internet connectivity. Similarly, in adjusted analyses, internet plan type could only explain a portion of reliable internet connectivity, with sociodemographic factors also being significantly associated. Variation may exist due to device limitations, limited data plans, or variable local service, each of which could leave an individual with an internet plan without internet access. These findings support the need to examine internet reliability through metrics that inquire specifically about actual internet connectivity experience, expectations of future internet connectivity, and the impact of sociodemographic experience rather than relying on metrics of internet plan use or device ownership alone.

Regarding geography or rurality, an important factor for delivering pediatric telemedicine in underserved communities [35,36], our analysis did not find statistical associations of reliable internet connectivity with geography (operationalized through metropolitan vs nonmetropolitan or 4 US Census regions). In prior research, Chagpar et al [30] found that internet use differed significantly by geographic region (ie, Northeast, Midwest, South, or West) among a large US population sample from the 2018 National Health Interview Survey. Paige et al [29] found that there were no significant differences in telemedicine use by rurality among a US random adult sample with a history of smoking tobacco. To our knowledge, our study was the first to assess the relationship between internet connectivity and geography among households with children.

We found 3 prior studies investigating the reliable internet connectivity metric, focusing specifically on having the internet working when people need it, with some key differences in questions assessing internet connectivity and in results. Katz et al [10] surveyed a US nationally representative sample of parents of school-aged children from low-income backgrounds and found that approximately 52% of these parents with home internet access reported that “their connection was too slow to do the things they wish to do online,” and 26% reported that they “feel that too many people share the same computer for them to have enough time on it, and 20% reported that their “Internet was cut off in the last year because of nonpayment.” Our rates of reliable internet connectivity, specifically among the lowest income bracket of respondents, approximated some of these findings (32%) but built on this work by estimating across households with all ages of children and all income brackets.

The Pew Research Center also assessed reliable internet connectivity through US adult self-reports of “problems with the speed, reliability or quality of their high-speed internet connections at home that make it hard to do the things they need to do online.” [37] In the Pew analysis conducted during the early COVID-19 pandemic, approximately 39% of higher-income and 60% of lower-income respondents reported that they often or sometimes had unreliable internet connectivity. Estimates from our survey (8% and 32%, respectively) were lower than these estimates, with possible reasons for these differences being our focus on families with children, excluding older respondents. Also, our survey fielding in 2022 meant that families were less reliant on the internet than they had been during the pandemic or the use of different questions.

Finally, Kan et al [7] measured reliable internet connectivity by surveying parents in a Midwest urban region, “How would you describe the internet that you most use at home?” with response options of “reliable, high-speed internet,” “reliable but slow internet,” “not reliable internet,” and “do not have internet at home.” They also inquired about parents’ worry about payment through the question, “Did any of the following happen over the past few months? … You worried about being able to pay for your internet connection,” with Yes or No response options [7]. These authors found that approximately 23% of parents reported unreliable high-speed internet, and 24% reported worry about paying for internet [7]. Whether internet reliability would have a similar prevalence outside of the Midwestern urban city was unclear; our results were relatively similar (19%).

Of note, these prior surveys asked about reliable internet connectivity experience in the past year [10,37], while others asked about the past few months [7] or did not specify a time duration at all [7]. There is a need for future research to derive consensus on the best questions that could adequately and accurately assess reliable internet connectivity as a separate construct, including specifying the definition, questions, response options, and time frame of such experience of reliable internet connectivity.

Even with a hypothetical validated metric on the reliable internet connectivity construct, there would be some families who choose not to engage with pediatric digital health services and exclusively prefer in-person care. Even among families without any internet plan or device ownership, we found that 75%‐79% still reported reliable internet connectivity in our unadjusted analysis. This finding might depend on how our 2 survey items on internet connectivity were framed to respondents, asking them if they worried about internet connectivity or experienced internet connection unreliability in the past 12 months. Some parents may have self-selected out of consistent engagement with digital technologies because they do not perceive the challenges of unreliable internet connectivity or hold negative attitudes towards technology use.

Eliminating pediatric digital health inequality for families is highlighted as a priority of the American Academy of Pediatrics [38]. Digital health inequality has been reported regarding inequitable access to the internet, technologies, or telemedicine for children [4,6,39] and adults [2,12,13,25,26]. At the individual or household level, key factors that contribute to digital health engagement include digital literacy, digital self-efficacy, technology access, and attitudes toward technology use [21]. These are all key factors to address, but we also propose the importance of reliable internet connectivity, which we found to be adjacent but not synonymous with digital technology access (ie, internet plan type and device ownership), acknowledging the need to account for sociodemographic factors as well.

Our findings illustrating digital health inequality in reliable internet connectivity were largely consistent with studies examining potential downstream consequences, revealing disparities in terms of internet use or telemedicine use [29,30]. To enhance access to reliable internet connectivity, our findings highlight the need for sustained investment in both individual interventions, such as programs to increase the affordability of and access to high-quality internet plans (eg, the federal government’s Affordable Connectivity Program [40], which recently ended, and Broadband Equity, Access, and Deployment Program [41]) and structural interventions (eg, expansion of internet infrastructure to low-income neighborhoods experiencing digital redlining) to reach marginalized populations. There is a need to consider designing technologies and connections that are accessible and usable for a wider range of sensory or cognitive abilities. Even with perfect household internet reliability, providing high-quality, accessible in-person clinical care remains an important investment for meeting pediatric health care needs.

Access to telemedicine plays a critical role in global emergency preparedness to ensure access to and continuity of care, mitigate infectious risks and costs, and close persistent population health gaps [42]. For example, during the COVID-19 pandemic, when in-person visits were especially strained, telemedicine use was leveraged to reduce pediatric mortality rates in Colombia [43], and improve chronic disease management in Kazakhstan [44], and it was deemed highly usable and satisfactory by patients in the Philippines [45]. Therefore, telemedicine access could be an important mechanism for ensuring health care access, especially in under-resourced global health contexts and during public health crises such as pandemics [43-45]. Our results suggest that understanding telemedicine access may require investigating not only device ownership and internet plans but also more experiential measures such as reliable internet connectivity in the home or the community.

Study limitations included that our items measuring the dependent variable of “reliable internet connectivity” (ie, internet worry or unreliable internet experience) were adapted from screening questions for other social determinants of health but were not validated outside the study. Future research is needed to validate the unreliable internet connectivity construct, including exploring how framing effects shape respondents’ answers. Our survey items asked specifically about worry or internet connection when they “really needed it,” which may underestimate the prevalence of reliable internet connectivity among families whose internet access is limited to the point that they do not even attempt to engage via the internet. The survey only classified residential areas as metropolitan versus nonmetropolitan (as opposed to a more granular assessment of rurality). Future studies should consider inquiring about rurality more specifically to assess the impact of rural and nonrural families on reliable internet connectivity. Our survey was administered in 2022, during the third year of the COVID-19 pandemic, which may affect parents’ perceptions and responses, and therefore, results may be less generalizable in a nonpandemic context.

This study was novel in that we assessed digital health disparities nationally for diverse families with youth younger than 18 years. Ensuring that US families with children have reliable internet connectivity could help reduce disparities in the use of pediatric digital health care, including telehealth use [28]. While no studies had measured the link between reliable internet connectivity and pediatric telehealth use, to our knowledge, one study found that delivering reliable internet connectivity at the community level led to significantly increased maternal telehealth use [46]. Future research should measure the relationship between reliable internet connectivity and pediatric telehealth use and outcomes.

### Conclusion

One-fifth of families with children experience unreliable internet connectivity in the United States. Unreliable internet connectivity was especially prevalent among populations known to also experience barriers to in-person health care, including individuals with disabilities, public insurance, racial and ethnic marginalized identities, and those who use languages other than English [2,7,10,12,13,27,37,47], potentially compounding access-related disparities through limited digital health care access [12]. Potential contributors to reliable internet connectivity included the internet plan type and quality of the devices (potentially related to family sociodemographic circumstances), local internet infrastructure (related to residence in rural or predominantly minority neighborhoods) [12,14,48], and ability to navigate connections and applications (related to sensory and cognitive abilities and digital health literacy) [49]. Because reliable internet connectivity is needed for the growing field of digital health care [50], this is a critical issue for equitable pediatric health care access and delivery.

## Supplementary material

10.2196/69304 Multimedia Appendix 1 Sensitivity analyses of multivariable logistic regression models

## Abbreviations

NIMHD: National Institute on Minority Health and Health Disparities
NORC: National Opinion Research Center
