# Supplementary material for: Measuring Reliable Internet Connectivity Among Families with Children: Secondary Analysis of a US National Survey
Source: J Med Internet Res. 2025 Aug 8;27:e69304. doi: 10.2196/69304 (PMC12333314; doi:10.2196/69304)
Supplement: Multimedia Appendix 1 [file jmir-v27-e69304-s001.docx]

**Appendix**

**Doan et al. Measuring reliable internet connectivity among families using pediatric digital healthcare: a US national survey**

**Table S1. Sensitivity analyses of alternative geography-related categories, reporting adjusted prevalence of respondents with reliable internet connectivity by technology access and sociodemographic variables**

|  | **Model 1** | | **Model 2** | |
| --- | --- | --- | --- | --- |
| **Characteristics** | **Adjusted proportions (95% CI)** | **Design-based  *P* value** | **Adjusted proportions (95% CI)** | **Design-based  *P* value** |
| **Household Internet Plan** |  | <.001* |  | <.001* |
| Mobile & Non-Mobile Plans | 0.86  (0.83-0.90) |  | 0.86  (0.83-0.90) |  |
| Mobile Plan-Only | 0.77  (0.70-0.85) |  | 0.77  (0.69-0.85) |  |
| Non-Mobile Plan-Only | 0.64 (0.54-0.74) |  | 0.63 (0.53-0.74) |  |
| None | 0.79  (0.50-1.00) |  | 0.80  (0.51-1.00) |  |
| **Household Device Ownership** |  | .237 |  | .313 |
| Smartphone & Non-Smartphone Devices | 0.82  (0.78-0.86) |  | 0.82  (0.78-0.86) |  |
| Smartphone Device-Only | 0.76  (0.69-0.83) |  | 0.77 (0.69-0.84) |  |
| Non-Smartphone Device-Only | 0.86  (0.79-0.94) |  | 0.86  (0.79-0.94) |  |
| None | 0.86  (0.67-1.00) |  | 0.86  (0.66-1.00) |  |
| **Respondent Education** |  | <.001* |  | <.001* |
| Less than High School | 0.79  (0.68-0.91) |  | 0.80  (0.68-0.91) |  |
| High School Graduate or Equivalent | 0.73  (0.65-0.81) |  | 0.74  (0.66-0.81) |  |
| Some College or Vocational School | 0.79  (0.74-0.83) |  | 0.79  (0.74-0.83) |  |
| Bachelor’s Degree | 0.87  (0.81-0.93) |  | 0.86  (0.81-0.92) |  |
| Postgraduate Study/Professional Degree | 0.95  (0.92-0.98) |  | 0.95  (0.91-0.98) |  |
| **Respondent Employment** |  | .003* |  | .005* |
| Yes | 0.85  (0.81-0.88) |  | 0.84  (0.81-0.88) |  |
| No | 0.75  (0.69-0.81) |  | 0.75  (0.69-0.81) |  |
| **Metropolitan Status** |  | .252 |  |  |
| Metropolitan | 0.80  (0.77-0.84) |  |  |  |
| Non-Metropolitan | 0.85  (0.79-0.91) |  |  |  |
| **US Census Region** |  |  |  | .313 |
| Northeast |  |  | 0.79 (0.69-0.90) |  |
| Midwest |  |  | 0.85  (0.79-0.90) |  |
| South |  |  | 0.79  (0.74-0.83) |  |
| West |  |  | 0.94  (0.79-0.89) |  |

Multivariable logistic models were applied with the above variables followed by predictive margins to generate adjusted proportions, 95% confidence intervals (CI), and *P* values. Results were starred (*) if considered statistically significant if *P*≤.05. This analysis accounted for survey design and sampling weights. Model 1 included technology access and sociodemographic variables, including metropolitan status. Model 2 included technology access and sociodemographic variables, including geographic region.

**Table S2. Sensitivity analyses of alternative race and ethnicity categories, reporting adjusted prevalence of respondents with reliable internet connectivity by technology access and sociodemographic variables**

|  | **Model 1** | | **Model 2** | |
| --- | --- | --- | --- | --- |
| **Characteristics** | **Adjusted proportions (95% CI)** | **Design-based  *P* value** | **Adjusted proportions (95% CI)** | **Design-based  *P* value** |
| **Household Internet Plan** |  | <.001* |  | <.001* |
| Mobile & Non-Mobile Plans | 0.86 (0.82-0.89) |  | 0.86 (0.82-0.89) |  |
| Mobile Plan-Only | 0.77  (0.70-0.85) |  | 0.78  (0.70-0.85) |  |
| Non-Mobile Plan-Only | 0.65 (0.54-0.75) |  | 0.65 (0.54-0.75) |  |
| None | 0.79  (0.51-1.00) |  | 0.79  (0.51-1.00) |  |
| **Household Device Ownership** |  | .190 |  | .187 |
| Smartphone & Non-Smartphone Devices | 0.82 (0.78-0.86) |  | 0.82 (0.78-0.86) |  |
| Smartphone Device-Only | 0.76 (0.69-0.83) |  | 0.76 (0.68-0.83) |  |
| Non-Smartphone Device-Only | 0.87 (0.80-0.94) |  | 0.87 (0.80-0.94) |  |
| None | 0.86  (0.67-1.00) |  | 0.86  (0.67-1.00) |  |
| **Respondent Education** |  | <.001* |  | <.001* |
| Less than High School | 0.81 (0.70-0.92) |  | 0.82 (0.71-0.93) |  |
| High School Graduate or Equivalent | 0.74  (0.66-0.81) |  | 0.75  (0.67-0.82) |  |
| Some College or Vocational School | 0.78 (0.73-0.83) |  | 0.79 (0.74-0.84) |  |
| Bachelor’s Degree | 0.86 (0.81-0.92) |  | 0.85 (0.79-0.92) |  |
| Postgraduate Study/Professional Degree | 0.95  (0.91-0.98) |  | 0.94  (0.91-0.98) |  |
| **Respondent Employment** |  | .003* |  | .002* |
| Yes | 0.85  (0.81-0.88) |  | 0.85  (0.81-0.88) |  |
| No | 0.75  (0.69-0.81) |  | 0.75  (0.69-0.80) |  |
| **Metropolitan Status** |  | .600 |  | .597 |
| Metropolitan | 0.81  (0.78-0.84) |  | 0.81  (0.78-0.84) |  |
| Non-Metropolitan | 0.83  (0.77-0.89) |  | 0.83  (0.77-0.89) |  |
| **Respondent Racial and Ethnic Marginalized Status** |  | .016* |  |  |
| Yes | 0.77  (0.73-0.82) |  |  |  |
| No | 0.85  (0.81-0.89) |  |  |  |
| **Respondent Race and Ethnicity** |  |  |  | .097 |
| Hispanic |  |  | 0.76 (0.70-0.82) |  |
| White, Non-Hispanic |  |  | 0.85  (0.81-0.89) |  |
| Black, Non-Hispanic |  |  | 0.76 (0.66-0.85) |  |
| Asian, Non-Hispanic |  |  | 0.85 (0.71-0.99) |  |
| Multiracial or Other, Non-Hispanic |  |  | 0.81  (0.70-0.92) |  |

Multivariable logistic models were applied with the above variables followed by predictive margins to generate adjusted proportions, 95% confidence intervals (CI), and *P* values. Results were starred (*) if considered statistically significant if *P*≤.05. This analysis accounted for survey design and sampling weights. Model 1 included technology access, sociodemographic, and racial and ethnic marginalized status variables. Model 2 included variables of technology access, sociodemographic, and individual race and ethnicity variables.
